# Hypereosinophilic syndrome presenting with multiple organ infiltration and deep venous thrombosis: A case report and literature review

**DOI:** 10.1097/MD.0000000000004658

**Published:** 2016-09-02

**Authors:** Su-jun Gao, Wei Wei, Jiang-tao Chen, Ye-hui Tan, Cheng-bao Yu, Mark Robert Litzow, Qiu-ju Liu

**Affiliations:** a Hematology section, Cancer Center, the First Hospital of Jilin University; b Department of Urology, The First Hospital of Jilin University, Changchun, Jilin, China; c Division of Hematology, Mayo Clinic, Rochester, Minnesota, USA; d Second Department of Cardiology, The First Hospital of Qiqihaer Hospital, Qiqihaer, Heilongjiang, China.

**Keywords:** corticosteroids, deep venous thrombosis, eosinophilia, hypereosinophilic syndrome, multi-organ infiltration, new oral anticoagulants, thrombosis

## Abstract

**Background:**

Hypereosinophilic syndrome (HES) can be fatal, particularly when eosinophils infiltrate vital organs and/or if extensive thrombosis develops. However there are no standard recommendations for the use of anticoagulant therapy of HES in the setting of thrombosis.

**Methods:**

We herein present a case of a 46-year-old female who presented with marked peripheral eosinophilia with symptoms of multi-organ infiltration and extensive deep venous thrombosis (DVT). In this case, evaluation was carried out before the diagnosis was established, and timely standard-dose corticosteroids combined with a new oral anticoagulant (NOAC) therapy were carried out.

**Results:**

These measures resulted in a rapid response and long-term disease control.

**Conclusion:**

Although there are no data to support which anticoagulant is preferred in this setting, this case indicates that the new oral anticoagulants may play an important role in the treatment of thrombosis in HES.

## 1 Introduction

Hypereosinophilic syndrome (HES) is characterized by peripheral blood eosinophilia (>1.5 × 10^9^/L) and associated organ damage. It is a rare, multisystem, heterogeneous syndrome with significant mortality. According to the Surveillance, Epidemiology and End Results database, the estimated morbidity is between 0.036 and 6.3/100,000,^[1]^ while the mortality is about 9.3%.^[2]^ Usually, HES was thought to affect mostly males, with a male:female sex ratio estimated at 9:1, especially in *FIP1L1-PDGFR*_*α*_ (*F/P*)-associated HES who are almost exclusively male. However, in other HES subgroups (lymphoid variant HES and idiopathic HES), the sex ratio seems closer to 1:1.^[3]^ Multiple organs can frequently be involved including the cardiac, respiratory, skin, gastrointestinal (GI), and neurological systems.^[4]^

The limited literature suggests that perhaps about 25% of patients develop thromboembolism, and 5% to 10% die as a result.^[5]^ It has been reported that the thrombosis can be cardiac, intra-abdominal, cerebral, cutaneous, and deep venous.^[4–9]^ Among them, deep venous thrombosis (DVT) is a rare, but often fatal complication of HES. Moreover, there is no consensus on whether anticoagulants should be routinely used in the setting of HES, and if used, which one or combination is the first choice. Some case reports and series have documented recurrent emboli despite adequate anticoagulation with warfarin or heparin but not with new oral anticoagulants (NOACs) or low molecular weight heparin (LMWH).^[10]^ Here, we report a successful use of rivaroxaban combined with prednisolone in the treatment of an idiopathic HES case presenting with extensive DVT and multi-organ infiltration. A systematic literature review is also presented.

## 2 Case report

A 46-year-old female was admitted to our emergency unit with a 1 month history of left upper abdominal pain, dry cough for 1 week, and a painful and swollen left lower extremity for 1 day. She was diagnosed with a benign gastric ulcer by gastroscopy and received anti-acid agents which were effective in relieving the abdominal pain, but not the cough or swelling. The patient had a history of congenital heart disease, but did not have a history of allergies or a remarkable family history. Physical examination showed pallor, with left lower leg swelling and tenderness.

The white blood cell count was elevated (32.6 × 10^9^/L) with 46.4% eosinophils (EOs) (absolute EO count 15.41 × 10^9^/L), hemoglobin of 10.3 g/dL, and thrombocytopenia of 7 × 10^9^/L. D-dimer was markedly elevated at 6055 μg/L. Clotting studies showed a decreased fibrinogen level (initially 1.13 g/L). However, the prothrombin time; activated partial thromboplastin time; activity of plasma protein C, protein S, and antithrombin III; and liver and kidney function tests were all within the normal range.

**Figure 1:**
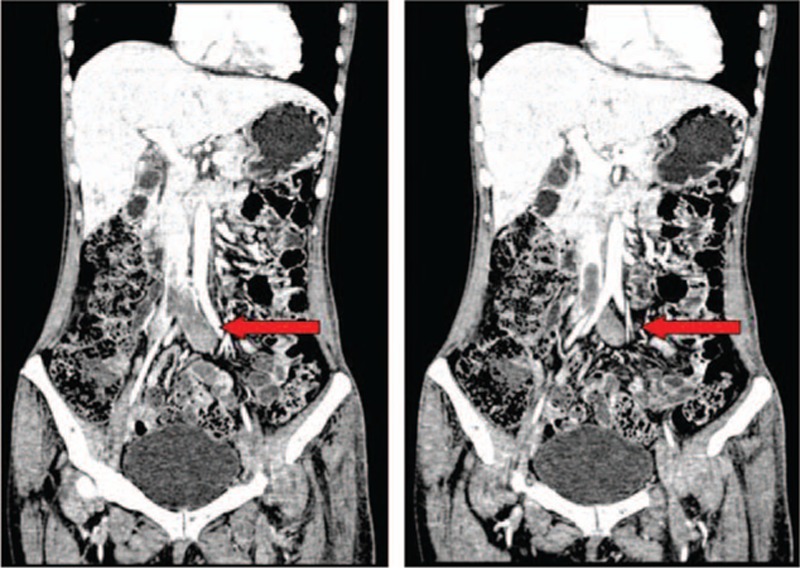
Abdominal–pelvic-enhanced computed tomography scan showing the thrombosis from inferior vena cava to the left tibial veins.

**Figure 2:**
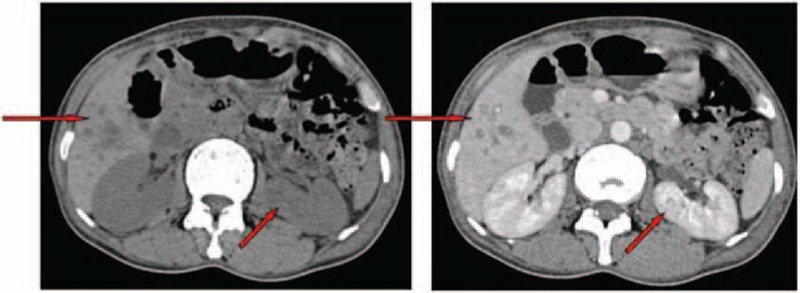
Abdominal–pelvic computed tomography scan showing multiple low density lesions in the liver and kidney.

**Figure 3:**
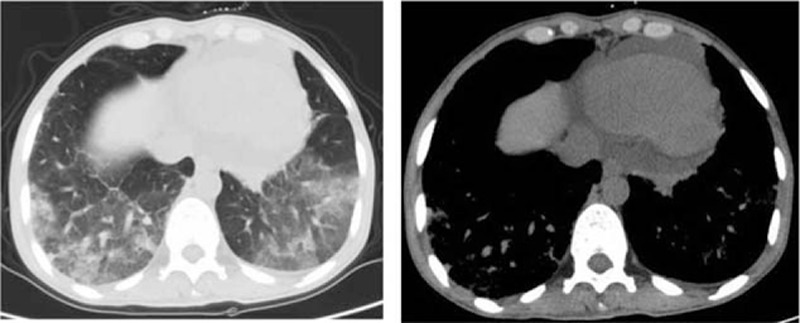
Chest computed tomography showing bilateral extensive consolidation at peripheral areas.

Ultrasound and 3-phase enhanced computed tomography (CT) demonstrated a large deep vein thrombosis from the inferior vena cava (IVC) to the left tibial veins (Fig. 1). The abdominal–pelvic CT scan also showed multiple patchy low-density lesions in the liver and kidney (Fig. 2), suggesting infiltration by EOs. Pulmonary CT scan showed bilateral extensive infiltration (Fig. 3, red arrows). An echocardiogram showed a congenital atrioseptal defect (secundum defects).

**Figure 4:**
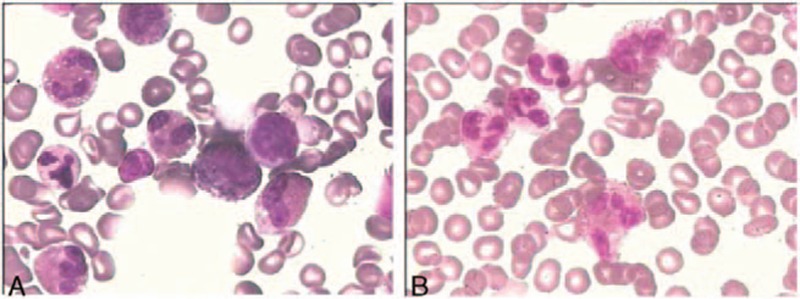
Hematoxylin–eosin stain of bone marrow (A) and peripheral blood cell (B), 400×, shows heavy infiltration of eosinophils.

Serum total immunoglobulin E was 242 IU/mL (reference range, <100 IU/mL). Bone marrow studies including aspiration cytology, biopsy, cytogenetic, and gene rearrangement analysis were done. The morphology revealed normal cellularity (35%) with no increase in blast cells, but with marked hypereosinophilia (61%) (Fig. 4). A normal karyotype (46, XY) was present. *JAK2 V617F*, *BCR-ABL*_*1*_, *FIP1L1-PDGFR*_*α*_, *PDGF*_*β*_, and *FGFR* were all negative as demonstrated by polymerase chain reaction (PCR) and FISH. There was no evidence of a B- or T-cell clone by marrow B- and T-cell receptor rearrangement analysis and flow cytometry. Stool and serum studies for parasitic infection and serum biomarkers for occult malignancy were negative. No evidence supported the existence of a connective tissue disease (anticardiolipid antibodies, antinuclear antibodies, and rheumatoid factors were negative).

**Figure 5:**
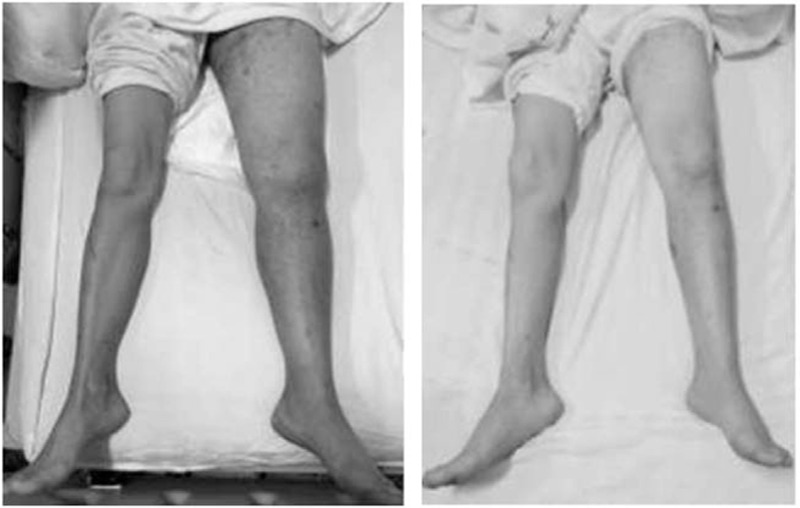
The swelling of left lower extremity that gradually regressed (after 11 days).

**Figure 6:**
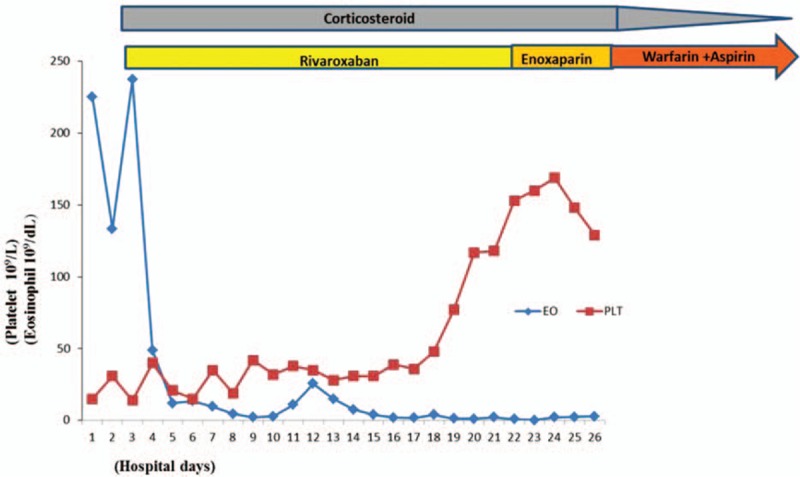
Timeline of treatment and changes of platelet and eosinophil counts.

The diagnosis of idiopathic HES was established based on exclusion of other causes. On day 3 of her admission, the patient was commenced on oral prednisone at a dose of 1 mg/kg/d and rivaroxaban 20 mg/d after a platelet transfusion. After 4 weeks, the steroid was tapered over the next 16 weeks. Her EOs count returned to normal range 2 weeks later after the use of prednisone, and the swollen extremity also progressively improved without increasing bleeding tendency (Fig. 5). At the 21st day of her hospitalization, recoveries of her platelet count (Fig. 6) and of the D-dimer level but not of the fibrinogen levels which continued decreasing (lowest 0.9 g/L) were noted. We ordered 6 units of cryoprecipitate transfusion and converted rivaroxaban to enoxaparin 40 mg bid. After 5 days, her fibrinogen level was normalized and remained stable. Then the patient was successfully anticoagulated with warfarin with discontinuation of enoxaparin and addition of aspirin. The patient was discharged on day 30 of her hospitalization and has remained healthy since then with follow-up of 1 year.

The patient provided written informed consent for the publication of these case details, and the consent procedure was approved by the ethic committee of the first hospital of Jilin University.

## 3 Discussion

In 1975, Chusid et al^[11]^ first proposed the 3 diagnostic criteria for HES. Our case fulfilled the diagnostic criteria of HES except for duration. However, the currently accepted definition of HES requires blood EO count greater than 1.5 × 10^9^/L on at least 2 occasions, ideally at minimum 1 month apart, and/or tissue eosinophilia that is considered excessive by the pathologist, and organ damage or dysfunction attributable to tissue EOs.^[12–14]^ However, there are controversies about how to define eosinophilic end organ damage. Evidence of EO-mediated tissue damage using methods other than histology is accepted with highly specific abnormalities that include thrombosis documented by imaging studies.^[15]^ Six clinical variants are designated according to the causes of HES: myeloproliferative HES, lymphocytic variant HES, overlap HES, associated HES, familial HES, and idiopathic HES.^[13–14]^ According to the above criteria of HES, our case can be provisionally diagnosed as idiopathic HES.

In our case, we also could not exclude the diagnosis of nonovert disseminated intravascular coagulation (DIC) according to the diagnostic criteria of the International Society on Thrombosis and Hemostasis.^[16]^ The changes of fibrinogen and D-dimer indicated the existence of consumptive coagulopathy. But after the recovery of D-dimer, the fibrinogen level continued decreasing which indicates the coexistence of primary fibrinolysis which may be caused by EO cationic protein (ECP).^[17]^ In another case report similar to ours, the authors controlled the coagulopathy with the addition of interferon-α (IFN-α) after failure of heparin.^[18]^

Usually HES involves at least 2 organ systems as it was also observed in our case (pulmonary, GI, kidney, liver, and vascular system), and according to the series reported by the Mayo Clinic these indicate that multi-organ disease may be a potential indicator of more severe disease and may possibly be a risk factor for a fatal outcome.^[2]^ Based on a compilation of case series and case reports, up to 25% of HES patients developed thrombotic complications.^[5]^ Moreover, venous thrombosis presents as the main clinical manifestation in some cases, while a poor prognosis is inferred to thrombosis itself.^[9]^ As such, the characteristics of this case mandated the swift initiation of treatment.

A full understanding of the mechanisms of eosinophilic disease pathogenesis remains elusive. However, a series of findings over the last 30 years has allowed for a better appreciation of the importance of cytokines, cytokines receptors and chemokines, particularly interleukein (IL)-5, IL-3, and granulocyte-macrophage clony-stimulating factor (GM-CSF), which all have important effects on trafficking, survival, degranulation, and activation of EOs.^[15]^ Activated EOs cause tissue and endothelial cell damage by a variety of mechanisms. EOs contain granules with some cationic cytotoxic proteins (such as ECP, major basic protein, EO peroxidase, and EO-derived neurotoxin), which can predispose to thrombosis by acting as platelet agonists, increasing vascular permeability, stimulating activation of factor XII, and reducing the anticoagulant actions of heparin.^[15,17]^ Recently, Cugno et al found higher tissue factor expression in patients with hypereosinophilic disorders which may contribute to increase the thrombotic risk.^[19]^ Overall, these studies point toward a combination of EO-mediated cytotoxicity toward endothelial cells, and a procoagulant state favored by hypereosinophilia. This combination of cell damage with resultant organ system dysfunction and increased thrombotic potential in HES patients may lead to fatal outcome.

With the improved understanding of the pathogenesis of HES, and the increasing ability to distinguish clonal HES from nonclonal HES, optimizing treatment outcomes becomes increasingly possible. Some authors suggest that treatment needs to be started when peripheral EO cells were more than 5000/μL or organ infiltration is documented.^[20]^ The general aims of HES treatment are to lower EOs count (below 1.0 × 10^9^/L) to alleviate clinical symptoms and to control damage to target organs. Steroids play a major role as the first-line treatment for idiopathic HES and other *F/P*-negative HES (*F/P*-positive HES should be treated with imatinib). The mechanism of corticosteroids is to interfere with the transcription of proinflammatory genes necessary for EO maturation, proliferation, migration, and chemoattraction. The median starting dose of prednisone was 1 mg/kg/d, but under severe conditions, higher dose can also be suggested. When EO count are lowered to normal levels and the symptoms are relieved, the dose can be tapered to 10 mg/d, and the duration of therapy can be variable, ranging from 2 months to 20 years. According to a retrospective analysis on HES treatment, 85% of these patients experienced a favorable response.^[21]^ In our case, we started treatment with prednisone as soon as we suspected the diagnosis of HES and documented a rapid and significant response. The second-line treatments of idiopathic HES are hydroxyurea, interferon-α, imatinib, and some new agents such as monoclonal antibodies targeting IL-5/IL-5Rα (mepolizumab) and anti-CD52 antibody (alemtuzumab), but thus far mepolizumab is not available outside of clinical trials.^[22]^

In our case, the most serious condition was the extensive thromboembolism involving the deep venous system. Since thrombosis is one of the serious complications of HES, the prevention and treatment of thromboembolism in these kinds of patients becomes urgent. Although the guidelines for preventing and treating thromboembolic events in patients with HES are not available, and whether to use anticoagulation or not is still controversial, for high-risk patients (i.e., with evidence of intracardiac thrombosis, DVT, or recurrent thromboembolic events), anticoagulation should be considered in the treatment of this potentially fatal complication.^[10]^

Before initiation of anticoagulation therapy, one of the contraindications needs to be considered. Usually, thrombocytopenia is one of contraindications for anticoagulation therapy with warfarin or heparin but not with LMWH or NOAC. It is more significant if we can estimate the bleeding risk guided by a prospectively validated clinical score, such as the RIETE score^[23]^ and the HEMORR2HAGES score.^[24]^ According to both of these scores, we evaluated the bleeding risk of our case, and the result did not indicate high risk. So, we did not place an IVC filter according to the current American College of Chest Physicians guidelines but proceeded with anticoagulation therapy.

There are no data to support the preferential use of heparin, LMWH, warfarin, or newer agents in thrombotic patients with HES. Commonly used agents include warfarin, heparin, or LMWH.^[4–9]^ However, thrombosis can be particularly difficult to manage. In some reports regarding HES patients, neither warfarin nor heparin avoided fatal outcome once thrombosis had occurred,^[8]^ but there are some successful cases with LMWH.^[4,25]^ Though the standard care of DVT is LMWH followed by a vitamin K antagonist, in our case we initially chose a Factor Xa inhibitor, rivaroxaban, in the setting of thrombocytopenia and suspected DIC, since some literatures indicate that a Factor Xa inhibitor may cause fewer major bleeding events than warfarin or LMWH.^[26–28]^ After the use of steroid and rivaroxaban, the patient's EO followed with platelet count recovered and the swollen extremity progressively improved, but the fibrinogen level remained low. We subsequently converted rivaroxaban to enoxaparin, and the fibrinogen levels remained stable after the cryoprecipitate transfusion. Since in HES patients about half (45%) of thrombosis were arterial,^[29]^ combination of anticoagulants with an antiplatelet agent may still have a role in preventing the recurrence of thrombosis in HES.

The recommended duration of anticoagulation is also unclear, but a number of reports suggested that discontinuation of therapy could be considered if eosinophilia was reliably controlled with no evidence of residual thrombus.^[10]^ In addition, although eosinophilia may be the primary cause of hypercoagulability in a patient with HES and a thromboembolic event, other potentially contributing factors, including immobility, acquired or genetic causes, hormone therapy, or other offending drug use, should also be considered and taken into account for minimizing the recurrence risk of life-threatening thromboembolic events.

In summary, we report successful control of HES and its serious complication of DVT in the setting of severe thrombocytopenia and suspected DIC by a combination of corticosteroids with NOAC bridged to LMWH, warfarin, and aspirin without increasing the bleeding tendency. One-year follow-up showed a complete remission. In this case report, we emphasize that thrombosis can be a significant cause of morbidity and mortality in patients with HES and indicate that beside rapid control of EO counts, effective anticoagulation treatment can play an important function in the control of this severe complication. Among those, NOAC can be one of the preferred choices. However, a large prospective clinical trial would be needed to determine which anticoagulant therapy is optimal in this setting.
